# The potential impact of wheat stem rust on global agricultural supply, demand, and food security, considering market interactions

**DOI:** 10.1371/journal.pone.0338959

**Published:** 2026-02-10

**Authors:** Benjamin Schiek, Athanasios Petsakos, Mesut Keser, Nicola Cenacchi, Timothy B. Sulser, Keith Wiebe

**Affiliations:** 1 International Center for Tropical Agriculture (CIAT), Palmira, Colombia; 2 Bioversity International, Rome, Italy; 3 International Center for Agricultural Research in the Dry Areas (ICARDA), Beirut, Lebanon; 4 International Food Policy Research Institute (IFPRI), Washington D.C., United States of America; Amity University, INDIA

## Abstract

Wheat stem rust, a fungal disease that can be highly devastating under the right environmental conditions, was reduced to non-economically damaging levels during the Green Revolution. However, it has reemerged as a global threat to wheat production due to the appearance of new virulent strains in Uganda in 1999 that have spread steadily to other geographic areas. Wheat experts warn that the disease could pose a catastrophic threat to the global wheat supply if not monitored. Considering the importance of wheat as a principal source of calories, nutrients, and farm income throughout the world, assessments of the potential impacts of the disease are urgently required in order to formulate an appropriate response. Published assessments so far vary widely in method and results, and generally focus on wheat production losses alone, without considering how markets may offset or aggravate impacts (spillover effects). Here we take an integrated assessment approach and examine a set of “what-if” scenarios to account for direct and indirect economic and food security impacts of wheat stem rust in various world regions over the years 2026–2050. The severity and frequency of epidemics is introduced into the modeling framework based on a survey of international wheat experts. The results suggest that global market incentives may offset the worst impacts of wheat stem rust in most affected areas via international trade. However, the market mechanism simultaneously precipitates considerable food insecurity in areas far from any epidemic, as farms in these areas reallocate resources from the domestic cereal market to the wheat export market, in response to price signals.

## Introduction

Wheat stem rust or black rust (hereafter “WSR”), caused by the fungus *Puccinia graminis* Pers. f. sp. *tritici*, posed a serious threat to global wheat production prior to the 1960s [1]. The threat subsided thereafter with the development of resistant cultivars during the Green Revolution. However, new virulent strains of the pathogen have emerged that threaten the most widely grown wheat (*Triticum aestivum*) varieties. The new strain, designated Ug99, was identified in Uganda in 1999 and spread to the rest of East Africa and Southern Africa, as well as Egypt, Yemen, and Iran [2,3]. More recently, the Ug99 strain group has been detected in Iraq [4]. In areas where variants of this group have spread, failure to apply countermeasures (plant newly resistant wheat varieties and apply fungicides) has resulted in yield loss of up to 80% on some fields [5]. Other newly evolving strains of WSR have caused significant yield loss in Europe [6,7], Russia [8], and Ethiopia, where 100% yield loss was reported on some fields [3].

A large international survey of crop experts to assess the current status of plant health management across a variety of crops and pathogens estimated an average WSR-induced 9% wheat yield loss across Africa South of the Sahara (SSA), and very little impact elsewhere [9]. However, high vulnerability to newly evolving strains of the pathogen is anticipated in regions where the disease has not yet spread [10–12].

To provide effective, targeted, and timely funding proportionate to the global threat posed by WSR, it is important to quantify the cost of inaction on this front. The few published estimates of WSR’s global economic and food security impacts vary substantially in method and magnitude. At the severe end, Fisher et al. [13] estimate that the wheat calories lost due to WSR would be enough to feed 0.2 to 1.4 billion people annually. Pardey et al. [14] critique this estimate, pointing out that it would imply an implausibly high loss of $8.7 to $61.2 billion USD annually at 2018 prices. Instead, they fit a probabilistic yield loss model on US wheat data, and then apply this model to the rest of the world to estimate a global wheat production reduction of 6 million metric tons (MT), or $1.28 billion USD, per year due to WSR. Chai et al. [15] subsequently update this analysis, projecting an average annual reduction of 0.9 to 9.9 million MT ($0.2 to $1.7 billion USD) over 2020–2050 due to WSR. While not as severe as the findings by Fisher et al. [13], these projections are still very alarming. More importantly, they are an order of magnitude above the current 9% yield loss in SSA estimated by Savary et al. [9].

Although these studies provide important contributions to our understanding of the potential global economic consequences of WSR epidemics and their implications for research, they focus on the assessment of production reductions directly resulting from WSR in afflicted regions, without considering WSR induced spillover effects in non-outbreak areas, and across other non-wheat food commodity markets. In other words, they omit the role that international trade and market mechanisms may play in offsetting and/or aggravating the direct biophysical impacts of the disease.

Economic reasoning suggests that crop production reductions, such as those caused by diseases like WSR, generate price signals that induce farmers in directly affected areas to reallocate land and resources to alternative crops not susceptible to the disease. They may also induce farmers in unaffected areas to reallocate land and resources to wheat for export to the affected areas. In directly affected areas, then, the market mechanism may, in some measure, offset domestic food security impacts—partly by wheat imports from unaffected areas, and partly by domestic reallocation of cropland and resources to alternative food commodities. At the same time, however, higher wheat prices will lead to changes in the food commodity bundle of consumers in both afflicted and unafflicted areas, with likely negative impacts on their food security status.

Assessments omitting treatment of this rather complex interaction of price signals and economic agency are thus prone either to understate or overstate WSR induced damages and food security impacts, depending on how the precise quantities hiding behind the phrase “in some measure” balance out. Capturing these interactions in a comprehensive way ideally requires a modeling framework built around global or regional multimarket models that simulate the economic equilibrium resulting from food production and consumption, combined with information from agronomic models which can simulate the complex biophysical conditions that affect crop growth and yield.

In the present article, we extend the discussion about the likely impacts of WSR on global wheat supply, demand, and food security by explicitly accounting for the potentially offsetting and/or aggravating effects of international multimarket mechanisms in response to WSR outbreaks. The severity and frequency of WSR yield losses in major wheat growing regions are introduced into the multimarket modeling framework based on expert opinion gathered from our extended network of wheat experts in the CGIAR. Results are presented in terms of best and worst case projections of the economic and food security impacts of WSR from 2026 to 2050, disaggregated by eight geographical regions. These best and worst case scenarios are defined with respect to a “No WSR” benchmark scenario, which represents a funding scenario sufficient to return WSR incidence and virulence to pre-Ug99 levels.

While similar multimarket models have become mainstream in foresight analysis and assessments of abiotic pressures related to climate change at global and regional scales [16–19], their use for understanding the potential economic damage by pests and diseases (P&D) under future scenarios has been scant. This is primarily due to major challenges faced in developing appropriate biophysical P&D models which can generate physical yield loss estimates required for economic assessments [20,21]. Among the few existing studies (to our knowledge), Wittwer et al. [22] modeled the economy-wide impacts of a hypothetical outbreak of wheat karnal bunt (caused by *Tilletia indica*) in Australia with a dynamic computable general equilibrium model, assuming a yield loss of 0.1% in the country’s wheat belt. Godfray et al. [23] performed a global partial equilibrium assessment of a hypothetical fungal disease affecting rice in Southeast Asia covering a single year, with yield losses based on expert opinion. More recently, Petsakos et al. [24] examined the potential impacts of banana Xanthomonas wilt in Africa by combining the same economic model used by Godfray et al. [23] with a field-level disease spread model and additional assumptions to translate spread into yield losses. The challenges of introducing explicit P&D modeling in global integrated assessment ensembles such as the one implemented here are discussed more broadly by Petsakos et al. [25]. Because of these challenges, here in lieu of explicit P&D spread modeling we introduce plausible WSR yield shocks and outbreak intervals based on expert input, following the lead of Godfray et al. [23].

In addition to extending the discussion about WSR impacts, then, the present work also aims to extend the multimarket economic modeling framework implemented in previous studies of other P&Ds by considering both spatial dynamics and the interannual variability of WSR outbreaks across different world regions, over a multi-year time horizon. Our geographical scope is also necessarily much broader, since newly evolving strains of WSR are now affecting, or soon likely to affect, nearly every major wheat producing region. It should be kept in mind that the present work represents an integrated assessment modeling approach to unanswered questions specifically regarding the role of multimarket mechanisms in mediating the direct impacts of WSR, at global scale. It does not explicitly address other relevant open questions regarding detailed pathology, co-occurring pathogens, or climate change-pathogen feedback linkages. This and other limitations are discussed at the end of the article.

## Materials and methods

### Expert survey on future yield losses to WSR

Information on the location, severity, and frequency of potential future WSR outbreaks used in this exercise is based on a survey of 11 international wheat stem rust experts and wheat breeders via a questionnaire sent over the period April-September of 2021. Respondents were asked to indicate likely national average wheat yield loss due to WSR and frequency of outbreaks over the next 10 years, within their geographical area of expertise and based on their experience during the last 10 years.

### Ethics statement

The 11 experts participating in the survey were selected based on their professional expertise in wheat science and pest management. Participation was voluntary, and all participants were informed of the study’s objectives and the intended use of the data. No sensitive personal data were collected. At the time of data collection, our institution did not have a formally consolidated Institutional Review Board (IRB). However, the current IRB has retrospectively reviewed the study procedures and determined that the research poses minimal ethical risk and conforms to the institution’s ethical standards for research involving human participants. All of the information collected is presented in Table 1.

**Table 1:** Summary of expert assessment of wsr outbreak frequency and related yield loss.

Outbreak frequency	Countries	Yield loss: Best case	Yield loss: Worst case
Every year	East Africa^1^	10%	20%
	Southern Africa^2^	0%	5%
Every 3 years	West Asia^3^	5%	10%
	North Africa^4^	5%	10%
Every 5 years	South & Central America^5^	5%	10%
	WSR-resilient Central Asia^6^	0%	5%
	WSR-vulnerable Central Asia^7^	5%	10%
	Russia	5%	10%
Every 10 years	Mediterranean Basin (Europe)^8^	5%	10%
	Western & Northern Europe^9^	0%	5%
	South Asia^10^	0%	5%
	USA	0%	5%
	Canada	5%	10%
	China	0%	5%
	Australia	5%	10%

^1^ East Africa = Ethiopia, Kenya, Uganda, Tanzania

^2^ Southern Africa = South Africa, Zambia, Zimbabwe

^3^ West Asia = Turkey, Israel, Lebanon, Syria

^4^ North Africa = Egypt, Libya, Morocco, Tunisia, Algeria

^5^ South & Central America = Argentina, Brazil, Uruguay, Paraguay, Chile, Peru, Colombia, Ecuador, Venezuela, Bolivia, Mexico, Honduras, El Salvador, Nicaragua, Panama, Costa Rica

^6^ WSR-resilient Central Asia = Afghanistan, Kyrgyzstan, Turkmenistan, Iran

^7^ WSR-vulnerable Central Asia = Uzbekistan, Tajikistan, Kazakhstan, Iraq

^8^ Mediterranean Basin (Europe) = Spain, Italy, Slovenia, Albania, Greece, Montenegro, Bosnia and Herzegovina, Croatia

^9^ Western & Northern Europe (excl. Spain) = France, Germany, Belgium-Luxembourg, UK, Ireland, Netherlands, Austria, Denmark, Switzerland, Poland, Sweden, Latvia, Lithuania, Estonia, Ukraine

^10^ South Asia = Bangladesh, India, Pakistan, Nepal, Afghanistan

### Bracketing the uncertainty, not predicting the future

As mentioned in the Introduction, future scenarios are examined in terms of a best and worst case. That is to say, the aim here is not to predict any single future scenario as being the most likely, but rather to identify plausible upper and lower bounds on what is likely to happen under plausible assumptions, given the very limited information that is available. Accordingly, the experts consulted for this study were asked to characterize expected national average yield loss in terms of a best case-worst case range, as opposed to a point estimate. Implicit in these scenarios are assumptions regarding the future incidence/severity of WSR and/or farmer adaptation to WSR (e.g., via fungicide or the planting of resistant cultivars). Hence, the best case scenario implies an optimistic outlook with respect to these elements, while the worst case scenario implies a relatively more pessimistic outlook. The expert assessment of outbreak frequency, and the lower and upper bounds on the anticipated WSR yield loss, are presented in Table 1.

### Geographical coverage

Respondents specifically covered Canada, Uruguay, Paraguay, Ethiopia, South Africa, India, Australia, and the Central/West Asia & North Africa region in general. This information was then synthesized and extrapolated to the remaining geographical areas included in Table 1, which were not explicitly covered by the respondents. In this way, the consultation ultimately covered most major wheat producing regions—68 countries in total. The only major wheat producing countries falling outside of the surveyed expertise were Romania, Bulgaria, Hungary, Czechia, and Slovakia. We were also unable to gather expert information (whether through direct consultation or literature) on a number of smaller but still relevant wheat producers, particularly Finland, Norway, Georgia, Armenia, Azerbaijan, New Zealand, and Japan. Together these omitted countries constituted less than 4% of global wheat production in 2020 [26], such that their exclusion does not significantly impact the multimarket equilibrium modeling.

Two experts consulted for the Central Asia & North Africa region characterized some Central Asian countries as more resistant to WSR than others, due to the presence of new cultivars and management practices. For this reason, the Central Asian countries in Table 1 are grouped into “WSR-vulnerable” and “WSR-resilient” categories. We note that the expert covering India expressed high confidence that WSR does not pose a major threat to wheat production in the South Asia region for the foreseeable future. This opinion is consistent with the near zero losses from WSR in the Indo-Gangetic plain (0.03%) estimated by Savary et al. [9]. It is also corroborated by the recent findings of Prasad et al. [27] who report extremely low WSR incidence in India over the period 2016–2022, due to unfavorable weather conditions for disease development, and because of recently developed wheat varieties which are resistant to many pathotypes of *Puccinia graminis*, including Ug99. Nevertheless, another expert consulted for this study expressed skepticism towards this opinion, especially in light of modeling by Meyer et al. [28] that suggests South Asia may be vulnerable to spore transmission from infected fields in Eastern Yemen. Because of this controversy, we have assumed a mild risk of WSR outbreak in South Asia, which translates to 0% yield loss for the best case and 5% for the worst case. Thus, the best case may be interpreted either as a reasonable forecast, for those who agree with the first expert, and the worst case yield loss assumption as an alternative “what if?” scenario, for those who agree with the second.

### Modeling the spatial distribution of outbreaks

The experts consulted for this study indicated the likely outbreak frequency for a given country or region (Table 1), but the spatial distribution of outbreaks at a given time remains to be determined. The spatial distribution is important because, as mentioned in the introduction, international market mechanisms can, to some extent, offset the food security impacts of outbreaks occurring in some regions but not in others. And, by the same token, this mitigating mechanism is rendered less effective as the number of regions where outbreaks occur at a given time increases. A scenario in which outbreaks occur in multiple regions at the same time may largely nullify the effectiveness of any offsetting market mechanisms.

Specifying a spatial outbreak schedule for WSR is a contemporary field of research which lies at the intersection of atmospheric physics and crop pathology and is accompanied by substantial uncertainty. Numerous airborne spore dispersal studies demonstrate a clear spatial correlation between outbreak regions with high atmospheric connectivity. That is to say, the fungus *Puccinia graminis* responsible for WSR can travel long distances by emitting spores which are then borne aloft on prevailing winds for up to a week before depositing in a new area [29,30]. For such aerial journeys to result in infection and spread of the disease, the airborne spores must: 1) survive the journey―i.e., remain within a specific range of environmental conditions; and 2) deposit in an area where a susceptible variety of wheat is under cultivation, and where environmental conditions facilitate WSR development [28,30]. In this way, it is thought that the WSR outbreak in Ethiopia in 2013/14 originally spread from Yemen [31]. It is also believed that *Puccinia graminis* may occasionally cross the Indian Ocean from Southern Africa to Australia [32,33], as well as the North Atlantic Ocean from North America to Europe [34]. Finally, the direction of airborne spore dispersal is not unique. Clear dispersal routes both out of East Africa into the Middle East, and from the Middle east into East Africa were reported by Meyer, Burgin, et al. [31].

**Fig 1:**
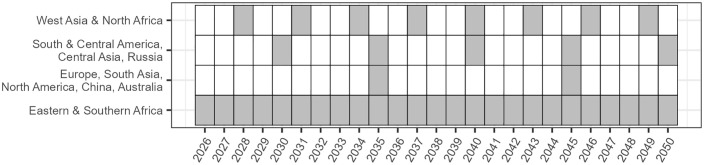
The 2026-2050 WSR outbreak schedule.

Because of the difficulty in defining a spatially and temporally explicit outbreak schedule for WSR, existing economic assessments have largely ignored the underlying biophysical dynamics. The probabilistic yield loss model employed by Pardey et al. [14] and Chai et al. [15] rests on the assumption that the spatial distribution of outbreaks in any given year is random and that outbreaks in any agroecological zone are independent from outbreaks in another zone. Similarly, the present assessment avoids delving into the mechanics of WSR outbreaks across space and time since it is beyond the scope of the intended analysis. Instead, among the numerous possible schedules, we select one that reflects 1) the region specific outbreak frequencies elicited from experts in Table 1, and 2) the high degree of spatial-temporal clustering suggested in the spore transport literature, such that years in which relatively few outbreaks occur are punctuated by relatively more severe years in which outbreaks occur across multiple regions. In this simulated schedule, presented in Fig 1, major outbreaks across multiple regions occur in the years 2030, 2035, 2040, 2045, and 2050. These five years are referred to collectively in the Results section below as the “global outbreak years”. The coincidence of outbreaks in these years is a result of the outbreak frequencies reported in each region by the consulted experts, and not due to any explicit modeling of outbreak dynamics.

### Global multimarket economic modeling of WSR outbreaks

The economic and food security impacts of the WSR outbreaks specified above were simulated with the International Model for Policy Analysis of Agricultural Commodities and Trade (IMPACT), developed and maintained at the International Food Policy Research Institute [35]. IMPACT is a global, partial equilibrium agricultural economic model which covers 62 commodity markets in 158 countries. It is linked to climate, crop, water, livestock, and fish models that together form an integrated assessment framework at global scale. IMPACT also includes post solution modules that generate a variety of food security indicators. (A more detailed description of IMPACT can be found in the model’s documentation [35]). Because of its capacity to simulate the economic equilibrium for a large number of crops across different world regions, and to output a broad range of indicators, IMPACT has been widely used for foresight analysis related to the global agricultural sector and is the most frequently used model in future food security assessments. Some of its applications include: (i) the analysis of climate change impacts on food production and food and nutritional security [18,19]; (ii) commodity analyses at different geographical scales [36,37]; and (iii) impact assessment for investments [38,39], agricultural research [40], and agricultural technologies [41].

Like other global economic models, IMPACT relies on a combination of standard scenario assumptions about climate and socioeconomic changes expected by 2050 [42]. Climate change assumptions take the form of Representative Concentration Pathways (RCP) that determine future carbon concentration and radiative forcing, which in turn drive future temperature increases [43]. Because commodity demand and supply are partly functions of future population, GDP, and technology growth, assumptions regarding these exogenous trends are bundled together in narratives called Shared Socioeconomic Pathways (SSP) [44,45].

Using data from the user-selected SSP-RCP, and combined with data from FAOSTAT and other databases, IMPACT solves for market clearing commodity prices at both the domestic and international level over the period 2020–2050. These solutions do not explicitly account for major short term disruptions in international trade (relatively frictionless trade). Domestic demand for agricultural commodities is calculated at the national level as a function of population, available income and prices. On the other hand, IMPACT calculates country level production of a given commodity as the economic yield times the harvested area. Farmers are thus assumed to adjust both land allocation and economic yield in response to price signals. This response is determined by country-specific supply elasticity parameters with respect to commodity price [35]. Yields solved by the model annually for each crop in each country are a function of producer output and input prices indices, water availability adjustments (based on results from the water model included in IMPACT), and climate shocks estimated with the DSSAT (Decision Support System for Agrotechnology Transfer) crop model [46] which is part of the IMPACT modeling suite. Yields also incorporate country- and commodity-specific exogenous yield growth factors representing baseline improvements in technology and management. (All parameters related to the IMPACT model, including those used in this study, can be found at https://github.com/IFPRI/IMPACT.)

Following Rosegrant et al. [18], for the present study we assume a combination of RCP7.0 and SSP2. This option suggests that we use the most extreme among the currently considered “realistic” climate change pathways [47] coupled with an assumed “middle-of-the-road evolution of future societal developments” with respect to climate change adaptation and mitigation challenges. SSP2 also posits moderate population growth, sustained economic development, and medium expected yield growth and input use intensity [48].

Data for RCP7.0 generated by the IPSL-CM6A-LR general circulation model (GCM) were obtained from the Inter-Sectoral Impact Model Intercomparison Project (ISIMIP3b) database [49]. This climate data is used as input to the DSSAT crop model to estimate future yields for all IMPACT crops, including wheat, under the selected RCP.

We first run a baseline scenario based on the SSP2-RCP7.0 assumptions, which we use as a reference for the analysis (the No-WSR scenario). Since the baseline wheat yield growth rate in IMPACT does not account for the reemergence of WSR, the No-WSR scenario may be interpreted as a benchmark scenario in which funding of wheat-related research is sufficient to return wheat systems to pre-WSR production levels. Following Petsakos et al. [24], we then introduce WSR yield losses by modifying IMPACT’s country-specific yield growth parameters mentioned above for both the best-and worst-case scenarios. Specifically, when the outbreak distribution schedule suggests an outbreak in a given region and year, we adjust the yield equation in that year for all countries in the region using the yield loss estimate obtained from the expert survey. We report results for supply, demand, and food security as the difference between the best and worst case WSR scenarios and the No-WSR benchmark scenario.

We note that the assumed frequency and severity of WSR yield losses are independent of the selected SSP-RCP scenario. In other words, the scenario simply provides the overarching socioeconomic assumptions for the analysis and the impact of abiotic climate stress on crop yields, as simulated by DSSAT, in the absence of WSR. This means that the climate assumptions under RCP7.0 do not affect the WSR yield losses which were estimated by the experts. For the same reason, although different SSP-RCP combinations are possible, and even alternative RCP implementations using other GCMs, our scenarios are specified only on the basis of the best and worst case yield losses.

## Results

Modeling results are presented below at the global and regionally disaggregated levels in terms of supply side, demand side, and food security impacts. The subject of impacts on international trade is cross-cutting, and is discussed in all three subsections to varying degrees. (See S1 Table in the Supplementary Information for the lists of countries included under each region heading. The raw model output is available at http://bit.ly/3KaJY66.)

### Supply side impacts

**Fig 2:**
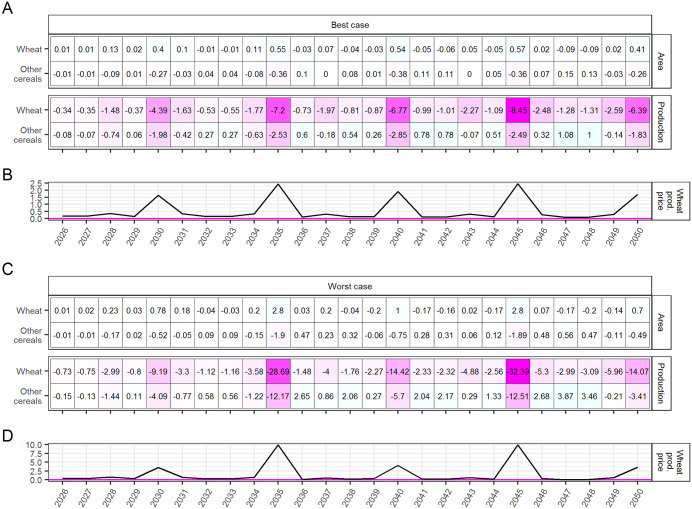
WSR impact on global wheat area (million hectares), production (million MT), and producer price (%), 2026-2050, expressed as the difference between the WSR and No-WSR scenarios. The impact on non-wheat cereal area and production is also included. Producer price is calculated as the value of production in 2005 USD divided by production in metric tons.

At the global level, the modeling projects that WSR yield loss will result in a higher wheat producer price relative to the No-WSR benchmark scenario (Fig 2, panels B and D), which in turn induces farms that are relatively unafflicted by WSR to allocate more area to wheat cultivation. As a result, global wheat area generally increases in years where outbreaks occur simultaneously in multiple regions (Fig 2, panels A and C, top). However, the modeling also projects that, on average, this increase in area will be insufficient to offset the decrease in wheat production directly caused by WSR (Fig 2A and 2C, bottom). The average of the projected annual reductions in wheat production relative to the No-WSR benchmark displayed in Fig 2 is 2.31–6.09 million MT (best case-worst case), or 0.25–0.65%. The mean reduction over the five global outbreak years of 2030, 2035, 2040, 2045, and 2050—i.e., the average peak reduction—is 6.64–19.75 million MT, or 0.71–2.11%.

Moreover, the new area allocated to wheat in response to the higher wheat price during the global outbreak years is mostly reallocated from other crops, primarily other cereals, thereby precipitating a decline in the production of non-wheat cereal crops as well (Fig 2A and 2C, bottom). The average of the projected annual decreases in other cereal production relative to the No-WSR benchmark displayed in Fig 2 is 0.30–0.76 million MT (0.01–0.03%), with an average peak of 2.34–7.57 million MT (0.10–0.31%) over the global outbreak years. The annual production of *total* cereals over 2026–2050 is projected to fall below the No-WSR baseline by an average of 2.61–6.84 million MT (0.26–0.68%), with an average peak of 8.98–27.33 million MT (0.81–2.42%) over the global outbreak years.

**Fig 3:**
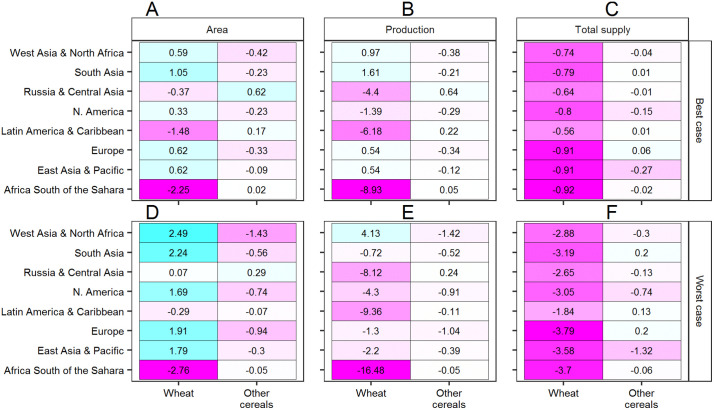
A regionally disaggregated focus on projected WSR impacts on area, production, and total supply (production + imports – exports) in 2035, expressed as the percentage difference between the WSR (best and worst case) and No-WSR scenarios.

To better illustrate the international supply side dynamics set in motion by WSR outbreaks, we present a regionally disaggregated view of impacts in a single global outbreak year, 2035, in Fig 3. Focusing on a global outbreak year is instructive because results are more pronounced. In 2035 of the present simulation, WSR outbreaks occur simultaneously in multiple “breadbaskets” across most of the globe except for West Asia & North Africa. In the best case scenario, South Asia is also exempt from the disease, and outbreaks in the East Asia & Pacific, Europe, North America, and Russia & Central Asia regions are partial, occurring in some parts of these regions but not in others. In the worst case scenario, outbreaks are prevalent everywhere within the afflicted regions, and South Asia is also afflicted. The modeling projects that biophysical yield loss due to outbreaks will generate price signals that result in one of two supply responses. Either farmers will find it more profitable to 1) evade the disease by reallocating land from wheat to alternative crops, primarily other cereals, or 2) compensate for WSR yield loss by reallocating land from other crops to wheat. (The information in Fig 3 is given in terms of magnitude in S1 Fig of the Supplementary Information.)

In the best case scenario, supply response 1 is prevalent in the Russia & Central Asia, Latin America & Caribbean, and Africa South of the Sahara regions, while supply response 2 is prevalent in the other five regions (Fig 3A). In the latter group, with the exception of N. America, the reallocation is sufficient to result in a projected increase in wheat production above the No-WSR baseline by a small percentage, at the cost of decreases in other cereal production below the No-WSR baseline by a small percentage (Fig 3B). In the former group, the economic reallocation of area from wheat to other cereals aggravates biophysical wheat yield loss, resulting in considerable projected decreases in wheat production below the No-WSR baseline, which are slightly offset by small projected increases in other cereal production above the baseline.

In the worst case scenario, the projected wheat price premium resulting from biophysical yield loss is so high that supply response 2 predominates almost everywhere (Fig 3D). The reallocation is most pronounced in the West Asia & North Africa, North America, Europe, and East Asia & Pacific regions. In worst case Russia & Central Asia, the area impact is positive for both wheat and other cereals, meaning that area is reallocated from non-cereal crops. Africa South of the Sahara is the one region exhibiting supply response 1 in the worst case scenario. Overall, area reallocations to wheat are insufficient to offset the direct biophysical impacts of WSR, resulting in projected decreases in wheat production below the No-WSR baseline by a considerable percentage, except in West Asia & North Africa, the one region where no outbreaks occur in this particular simulation (Fig 3E).

In both the best and worst case scenarios, increases in wheat area in response to the wheat price premium are insufficient to compensate for biophysical yield loss, resulting in considerable projected reductions in total wheat supply below the No-WSR baseline (Fig 3C and 3F). In the South Asia, Latin America & Caribbean, and Europe regions, the wheat supply deficit is slightly offset by a projected increase in the supply of other cereals above the baseline. In the other five regions, however, the wheat supply deficit is aggravated by projected decreases in the supply of other cereals below the baseline.

### Demand side impacts

**Fig 4:**
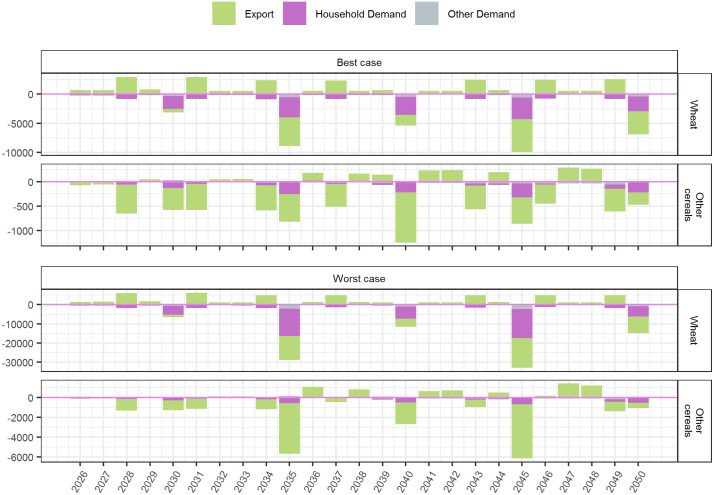
Projected global WSR impact on export, household, and other demand for wheat and other cereals, 2026-2050, expressed as the difference in 1000 MT between the WSR (best and worst case) and No-WSR scenarios. “Other demand” = livestock feed, seed, and intermediate demand.

Demand side WSR impacts reflect interactions with, and offer additional insight into, the supply side impacts presented above. As mentioned, production losses due to WSR are projected to precipitate numerous shifts in wheat and non-wheat export demand. Globally, annual export demand for wheat is projected to rise moderately above the No-WSR benchmark during moderate outbreak years, and to fall sharply below it, together with household demand, during global outbreak years. The average projected annual decrease in household wheat demand below the No-WSR baseline over 2026–2050 is 0.84–2.22 million MT (0.16–0.41%), with an average peak of 3.01–9.11 million MT (0.55–1.68%) over the global outbreak years. The impact on demand for other cereals exhibits a similar pattern, albeit to a lesser degree. The global year-by-year impacts on key demand categories are displayed in Fig 4.

**Fig 5:**
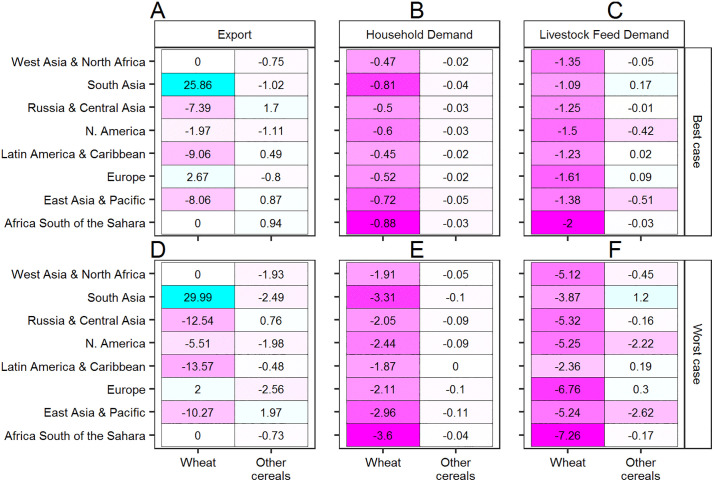
A regionally disaggregated focus on the projected WSR impact on export, household, and livestock feed demand for wheat and other cereals in 2035, expressed as the percentage difference between the WSR (best and worst case) No-WSR scenarios.

To better illustrate these demand side commodity market dynamics, we present a regionally disaggregated view of impacts in the global outbreak year of 2035 (Fig 5). Generally speaking, wheat export demand is projected to fall considerably below the No-WSR benchmark in most regions except for South Asia and Europe, where comparatively lower WSR incidence and severity results in a trade advantage (Fig 5A and 5D). In the best case scenario, the lower wheat exports are slightly offset by a small projected increase in exports of other cereals above the No-WSR baseline—except for North America, where lower production of non-wheat cereals due to reallocation of land to wheat results in lower export demand for all cereals. In the worst case scenario, the global wheat deficit is aggravated by an accompanying fall in exports of other cereals below the No-WSR baseline. (The information in Fig 5 is given in terms of magnitude in S2 Fig of the Supplementary Information.)

While trade may offset sharp reductions in severely afflicted regions, then, it achieves this by precipitating domestic cereal supply reductions in comparatively less afflicted regions (Fig 5B and 5E). Notably, the decrease in household consumption of wheat below the No-WSR benchmark in South Asia and Europe—regions with comparatively low WSR incidence and severity—is projected to be comparable to or worse than the decrease in regions with high WSR incidence and severity. South Asia, in particular, is projected to have the highest magnitude decrease, and second highest percentage decrease, in household wheat demand, despite its presumed biophysical resilience to WSR in this simulation. Livestock feed demand is likewise projected to fall below the No-WSR baseline in all regions by a considerable percentage, suggesting downstream food security repercussions beyond the immediate reduction in cereal consumption (Fig 5C and 5F).

### Food security impacts

The multimarket supply and demand analysis so far suggests that international trade may partly offset the global wheat deficit relative to the No-WSR baseline in any single region via increased wheat export demand from comparatively unafflicted regions, and, to a lesser degree, increased export demand for other cereals from comparatively afflicted regions. But these mitigating market mechanisms fall far short of completely offsetting the deficit, resulting in lower household consumption of both wheat and other cereals in all regions, afflicted and unafflicted, relative to the No-WSR baseline. What does this mean in terms of food security? The IMPACT model estimates population at risk of hunger (similar to FAO’s prevalence of undernourishment (PoU)) using a formula originally developed by Fischer et al. (2005), which is based on an empirical relationship observed between hunger and food availability in caloric terms.

**Fig 6:**
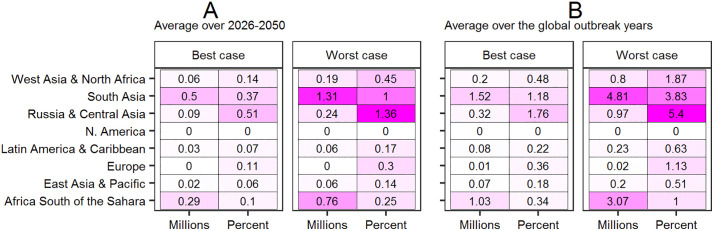
Projected annual difference in population at risk of hunger between the WSR and No-WSR scenarios, in terms of millions of people and percent. Panel A presents the average best and worst case difference taken over 2026-2050; while panel B presents the average best and worst case difference taken over the subset of global outbreak years (2030, 2035, 2040, 2045, and 2050).

Globally, the annual number of people at risk of hunger under WSR is projected to rise above the No-WSR benchmark by an average of 0.99–2.64 million (0.17–0.47%), with an average peak of 3.23–10.11 million people (0.58–1.81%) over the global outbreak years. In the regionally disaggregated view, the projected average percentage increase is most pronounced in Russia & Central Asia, a major wheat producing region with moderate to high WSR incidence and severity (Fig 6). The second most pronounced average percentage increase, and most pronounced in terms of numbers, is projected in South Asia. This is a remarkable result considering that South Asia is presumed to have the lowest WSR incidence and severity of all regions in the modeled scenarios. Considerable increases in the population at risk of hunger are also projected in Africa (north and south) and West Asia.

## Discussion and conclusion

Existing WSR impact assessments focus on global aggregate production reductions directly caused by the pathogen, without considering the “knock on” effects the direct shock has on other commodity markets in directly afflicted areas, and on commodity markets in other regions beyond the directly afflicted areas. To redress this gap, here we explicitly model the economic repercussions of the purely biophysical yield loss caused by WSR on international commodity markets. This allows us to examine the role that markets play in aggravating or offsetting the production reductions directly caused by WSR, and to report results in a spatially disaggregated way.

The results of the modeling exercise suggest that the occurrence of WSR may activate market reallocation mechanisms—via price signals—that can offset the wheat supply reduction in directly afflicted regions to varying degrees, while also inducing supply reductions in comparatively unafflicted regions, depending upon the incidence and severity of outbreaks. The reallocation mechanisms are twofold: First, there is a reallocation of land and resources—from wheat to other commodities in regions with high presumed WSR incidence and severity, and from other commodities to wheat in the comparatively unafflicted regions. And, secondly, there is a reallocation of production—from the export to the domestic market in afflicted regions, and from the domestic to the export market in comparatively unafflicted regions.

These reallocation mechanisms partially mitigate the intensity of the WSR wheat supply reduction in afflicted countries by redistributing the deficit more evenly across all countries participating in the international commodity market. In particularly bad outbreak years, these mechanisms may serve to avert catastrophic cereal supply reductions in directly afflicted areas, but at the cost of inducing significant reductions and underserved domestic cereal markets in regions that are far from any outbreak. On the one hand, this is reassuring since it suggests that market forces will insulate WSR-vulnerable countries from the worst biophysical consequences of the disease. On the other hand, it also means that no country is safe from WSR induced supply reductions. Countries not directly afflicted by WSR are indirectly susceptible to WSR induced supply reductions through international trade.

In numbers, the present exercise projects that WSR can lead to a global average annual wheat supply reduction of 2.31–6.09 million MT over the years 2026–2050, relative to the No-WSR benchmark. This is much greater than the global reduction implicit in Savary et al. [9], and on the same order of magnitude of the projections by Pardey et al. [14] and Chai et al. [15]. More precisely, the worst case estimate reported here is in rough agreement with the reduction projected by Pardey et al. [14], but about 3 million MT lower than the upper bound projected by Chai et al. [15]. The best case estimate reported here is considerably lower than Pardey et al.’s estimate, and about 1.5 million MT higher than Chai et al.’s lower bound. Accounting for reductions in non-wheat cereal production due to reallocation of land from other cereals to wheat, the present exercise estimates that total cereal production may fall below the No-WSR baseline by an annual average of 2.61–6.84 million MT, with an average peak of 8.98–27.33 million MT over the five global outbreak years.

Whereas existing WSR impact assessments focus exclusively on the supply side, the present study moves beyond this to also consider demand side and food security impacts. The results of this exercise project a global annual average decrease in household wheat consumption of 0.84–2.22 million MT over the years 2026–2050, relative to the No-WSR benchmark, with an average peak of 3.01–9.11 million MT over the global outbreak years. In terms of food security impacts, the total number of people at risk of hunger over 2026–2050 is projected to be an average of 0.99–2.64 million per year higher than under the No-WSR benchmark, with an average peak of 3.23–10.11 million per year during the global outbreak years.

In the spatially disaggregated view, the worst food security impacts are projected in South Asia, a region with comparatively low anticipated WSR incidence and severity in the outbreak scenario considered here. Regardless of one’s opinion on South Asia’s biophysical vulnerability to WSR, this is an instructive result, as it demonstrates that even biophysically resilient regions may be vulnerable to WSR induced food insecurity, and thus have a vested interest in eradicating the disease.

The ex-ante impact assessment presented here rests upon a few tacit assumptions beyond the SSP and RCP made explicit in the Materials and Methods section. First, although the impact of climate change on crop yields is captured by the crop models linked to IMPACT, we do not explicitly consider the combined effect of both climate and WSR on crop yields. Instead, we rely on expert opinion to quantify potential yield losses to WSR in the future and in different regions. Similarly, we do not model how the aerial transfer of spores may be affected by climate change. While previous research can provide information on the risk of climate change exacerbating WSR infection at different scales [32], using such findings in integrated assessment models like IMPACT requires translating infection risks into productivity shocks. Combined crop-disease models that can potentially cover this requirement are still under development [21].

Secondly, given that WSR is prevalent in almost all wheat growing regions, the yield shocks provided by experts reflect their (subjective) opinion on the additional loss incurred by the disease. Implicit in this estimate is the response of farmers and other stakeholders to the disease, and by consequence the overall level of plant health management to 2050. Possible improvements in plant health protection over time, and/or the role of policies in stabilizing prices, are not explicitly modeled—although they are considered indirectly through the best and worst case assumptions. Future research could explore alternative assumptions, accompanied by the development of additional scenarios regarding the level of plant health management and policy interventions, so long as it does not cause a “curse of dimensionality” problem that would render the analysis intractable.

Thirdly, the present study suspends from consideration other relevant pathogens besides WSR affecting wheat and other crops. While such ceteris paribus assumptions are typically necessary to avoid attribution problems, WSR is clearly not the only exogenous shock of concern over the next 25 years. The study by Savary et al. [9] identifies dozens of pathogens likely to afflict wheat and other key crops in the coming decades. It may be worthwhile, then, to examine scenarios in which multiple exogenous shocks occur simultaneously—although, generally speaking, one can expect the same broad result presented here, i.e., that markets will tend to moderate the severity of production loss in any single location by spreading it to other regions of the world. And, as a general rule, the realism gained by including more shocks comes at the cost of reduced insight into the contribution of any single shock to their collective impact. The study by Chai et al. [15] considers four fungal wheat diseases in addition to WSR, and is thus an important step in this direction. However, as noted above, Chai et al. do not account for multimarket interactions as we do here. A multimarket, multi-peril approach would have to overcome the challenges discussed by Petsakos et al. [25].

Finally, the multimarket equilibrium model assumes that markets react to WSR outbreaks instantaneously, whereas in reality one would probably expect a short lag between an outbreak and reallocation of farmland in response to price signals. This is a problem common to all equilibrium models, which, by definition, assume annual market clearing. We acknowledge that a market disequilibrium is likely to occur in the short term if many world regions are subject to yield shocks simultaneously, which means that equilibrium models may not always be the best tool to simulate such shocks in commodity supply [23].

Future research on WSR effects can relax the assumptions enumerated above by considering the incidence of multiple plant pathogens in both wheat and other staple crop production. This might be achieved, for example, by combining elements of the “multi-peril” study by Chai et al. [15] with the integrated assessment modeling approach pursued here. As such modeling ensembles progress and overcome their current limitations, it may also be interesting to explore how models explicitly linking disease dynamics and severity to climate change can be integrated into multimarket frameworks like the one used here [25]. Redressing the assumption of instantaneous market reactions may require a more nuanced modeling approach, and/or interpretation of results—one which takes more explicit account of, for example, the role of stocks in mitigating supply shocks, as well as the role of agroclimatic advisory services in alerting farmers to the likelihood of outbreaks.

## Supporting information

S1 Fig A re gionally disaggregated focus on projected WSR impacts on area (1000 hectares), production (1000 MT), and total supply (production + imports – exports, 1000 MT) in 2035, expressed as the difference between the WSR (best and worst case) and No-WSR scenarios. (TIFF)

S2 Fig A regionally disaggregated focus on the projected WSR impact on export, household, and livestock feed demand for wheat, other cereals, and non-cereal crops in 2035, expressed as the difference in 1000 MT between the WSR (best and worst case) and No-WSR scenarios. (TIFF)

S1 Table Region groupings used for the presentation of WSR impacts in the Results section. (DOCX)
