# Supplementary figures and images for: The potential impact of wheat stem rust on global agricultural supply, demand, and food security, considering market interactions
Source: PLoS One. 2026 Feb 10;21(2):e0338959. doi: 10.1371/journal.pone.0338959 (PMC12890143; doi:10.1371/journal.pone.0338959)

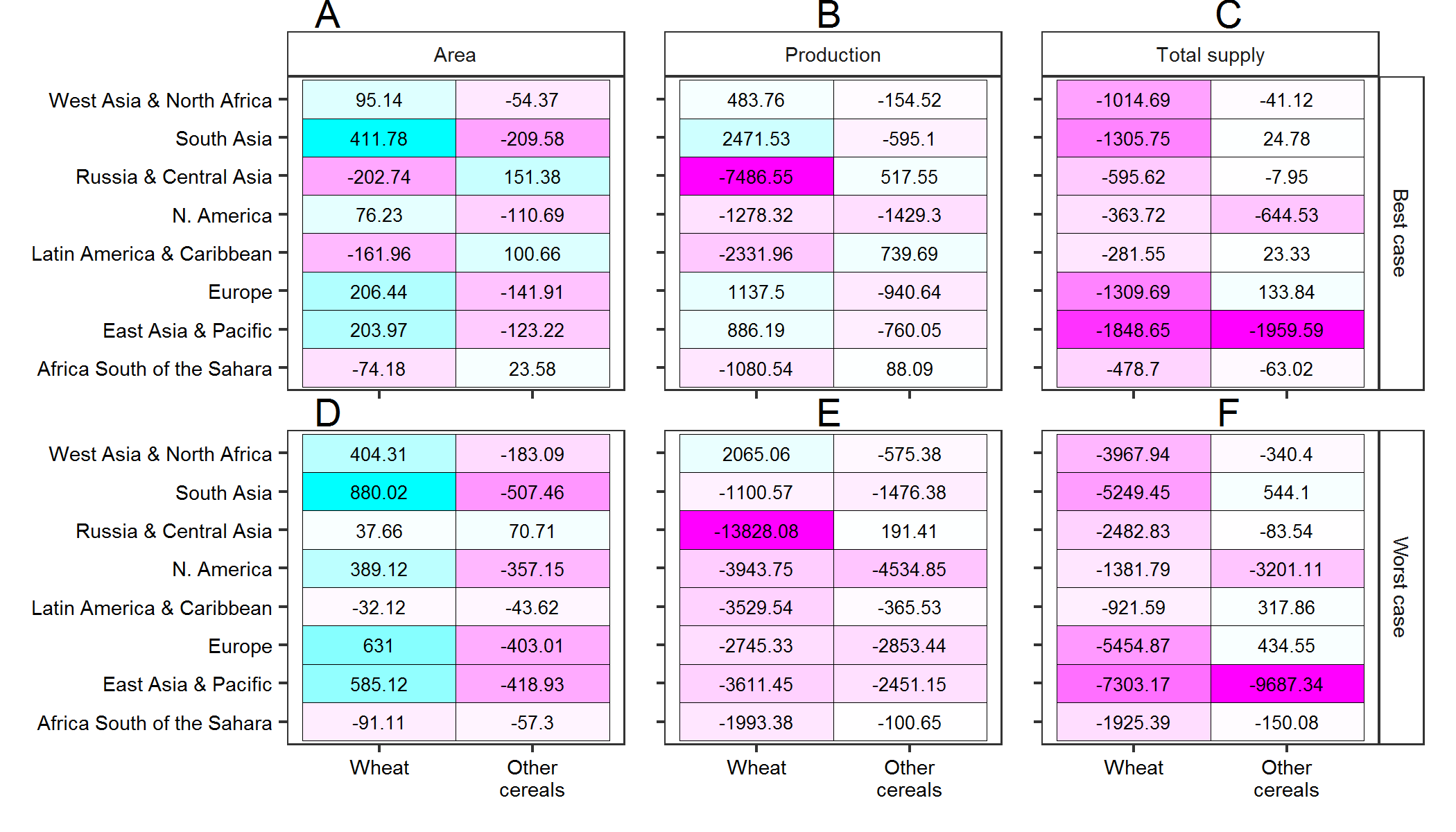

Supplement: S1 Fig — (TIFF) [file pone.0338959.s001.tiff]

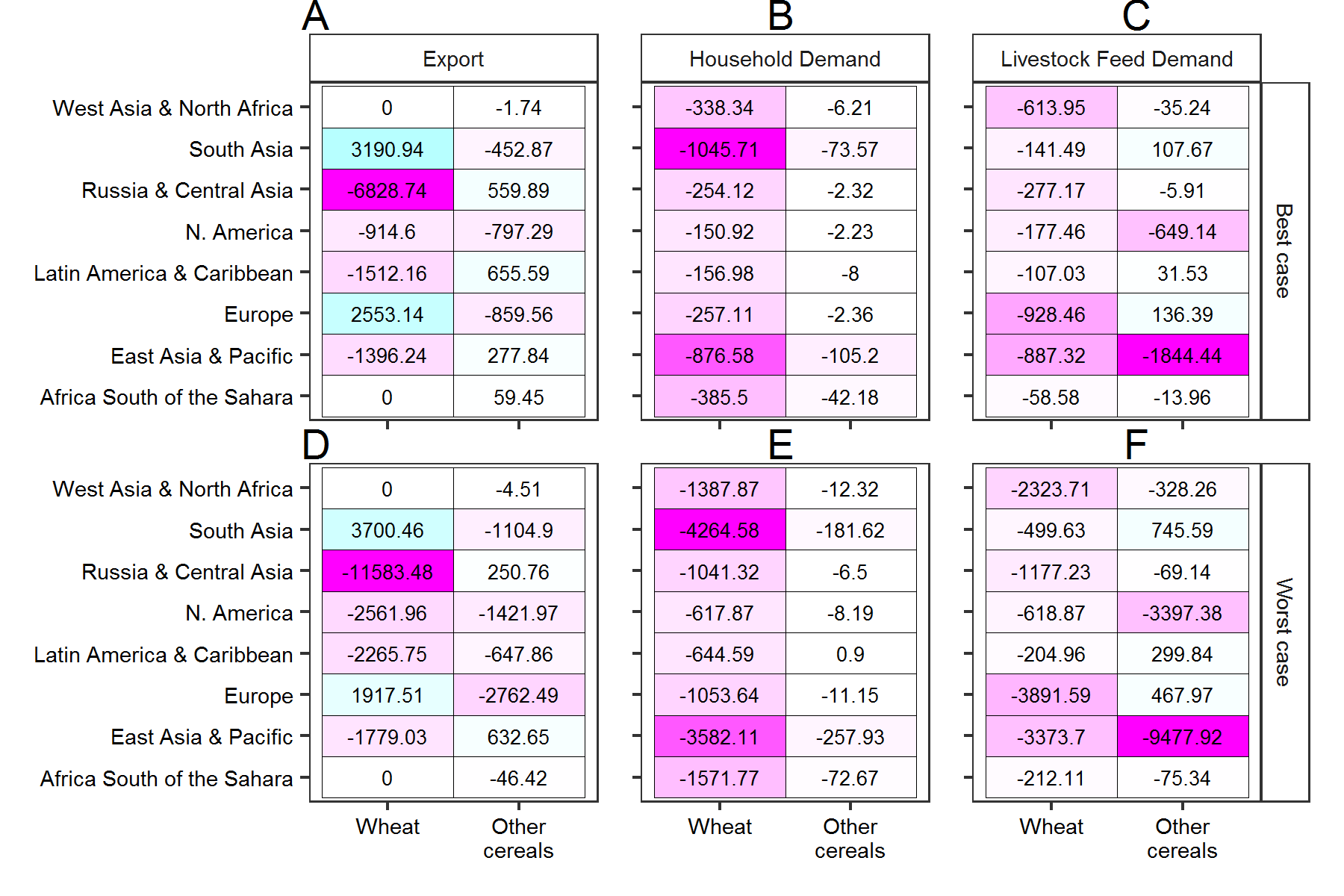

Supplement: S2 Fig — (TIFF) [file pone.0338959.s002.tiff]
