# Supplementary material for: The potential impact of wheat stem rust on global agricultural supply, demand, and food security, considering market interactions
Source: PLoS One. 2026 Feb 10;21(2):e0338959. doi: 10.1371/journal.pone.0338959 (PMC12890143; doi:10.1371/journal.pone.0338959)
Supplement: S1 Table — (DOCX) [file pone.0338959.s003.docx]

| S1 Table. Region groupings used for the presentation of WSR impacts in the Results section | |
| --- | --- |
| Russia & Central Asia | Afghanistan, Armenia, Azerbaijan, Georgia, Iran, Kazakhstan, Kyrgyzstan, Russia, Tajikistan, Turkmenistan, Uzbekistan |
| Africa South of the Sahara | Angola, Burundi, Benin, Burkina Faso, Botswana, Central African Rep., Ivory Coast, Cameroon, DRC, Congo, Djibouti, Eritrea, Ethiopia, Gabon, Ghana, Guinea, Gambia, Bissau, Equatorial Guinea, Kenya, Liberia, Lesotho, Madagascar, Mali, Mozambique, Malawi, Namibia, Niger, Nigeria, Other Atlantic, Rwanda, Sudan, Senegal, Sierra Leon, Somalia, Swaziland, Chad, Togo, Tanzania, Uganda, South Africa, Zambia, Zimbabwe |
| Europe | Albania, Austria, Bulgaria, Belarus, Baltic States, Luxembourg, Switzerland, Cyprus, Czech Republic, Germany, Denmark, Finland, France, Greece, Croatia, Hungary, Ireland, Iceland, Italy, Moldova, Netherlands, Norway, Other Balkans, Poland, Portugal, Romania, Spain, Slovakia, Slovenia, Sweden, UK, Ukraine |
| Latin America & Caribbean | Argentina, Belize, Bolivia, Brazil, Chile, Colombia, Other Caribbean, Costa Rica, Cuba, Dominican Republic, Ecuador, Guyanas, Guatemala, Honduras, Haiti, Jamaica, Mexico, Nicaragua, Panama, Peru, Paraguay, El Salvador, Uruguay, Venezuela |
| East Asia & Pacific | Australia, China, Fiji, Indonesia, Japan, Cambodia, South Korea, Laos, Myanmar, Mongolia, Malaysia, New Zealand, Other Indian Ocean, Other Pacific Ocean, Other Southeast Asia, Philippines, Papua New Guinea, North Korea, Solomon Islands, Thailand, Timor L'Este, Vietnam, Vanuatu |
| South Asia | Bangladesh, Bhutan, India, Sri Lanka, Nepal, Pakistan |
| N. America | Canada, USA |
| West Asia & North Africa | Algeria, Egypt, Iraq, Israel, Jordan, Lebanon, Libya, Morocco, Mauritania, Palestine, Rest of Arabia, Saudi Arabia, Syria, Tunisia, Turkey, Yemen |
